# Rivaroxaban vs. warfarin for the treatment and prevention of venous thromboembolism: A meta-analysis

**DOI:** 10.3389/fsurg.2023.1086871

**Published:** 2023-04-17

**Authors:** Zhuang Liu, Dan Song, Liang Wang, Changfeng Wang, Jie Zhou, Jiali Sun, Lei Guo

**Affiliations:** Department of Vascular Anomalies and Interventional Radiology, Jinan Children's Hospital, Children's Hospital Affiliated to Shandong University, Jinan, China

**Keywords:** rivaroxaban, venous thromboembolism, warfarin, treatment, prevetion

## Abstract

**Background:**

Anticoagulant treatment is used to treat and prevent venous thromboembolism (VTE). However, the relative effectiveness of newer anticoagulants vs. warfarin has not been appraised.

**Objective:**

The aim was to evaluate the safety and efficacy of rivaroxaban for VTE in comparison to warfarin.

**Materials and methods:**

From January 2000 until October 2021, all related studies were collected by EMBASE, the Cochrane Library, PubMed and Web of Scienceand. During the review process, two reviewers independently analyzed the included studies, including quality evaluation, screening and data extraction. We focused on VTE events as our primary outcomes.

**Results:**

In total, 20 trials were retrieved. These studies involved 230,320 patients, of which 74,018 received rivaroxaban and 156,302 received warfarin. Compared with warfarin, the incidence of VTE in rivaroxaban is significantly lower (risk ratio (RR) 0.71, 95% confidence interval (CI) [0.61, 0.84]; *P* < 0.0001, random effect model), and signiﬁcantly reduced major [RR: 0.84, 95% CI (0.77, 0.91); *P* < 0.0001, fixed effect model] and nonmajor [RR: 0.55, 95% CI (0.41, 0.74); *P* < 0.0001, fixed effect model] bleeding. No signiﬁcant differences in all-cause mortality between the two groups [RR: 0.68, 95% CI (0.45, 1.02); *P* = 0.06, fixed effect model].

**Conclusion:**

Rivaroxaban significantly reduced the incidence of VTE compared to warfarin in this meta-analysis. In order to verify these findings, larger sample sizes are required in well-designed studies.

## 1. Introduction

VTE includes deep vein thrombosis, central venous catheter-associated thrombosis, and pulmonary embolism, is a potentially life threatening condition (1, 2).

VTE can be prevented and treated with anticoagulant therapy (3); There have been several parenteral anticoagulants used historically, including low molecular weight heparin, unfractionated heparin, fondaparinux and warfarin (4). As a commonly used anticoagulant drug, warfarin has some drawbacks: the INR needs to be checks while taking the drug and certain foods increase the risk of bleeding, such as spinach, green tea and goji berries (5). Warfarin also has the potential to cause skin necrosis [Patel NB, Jain G. Warfarin induced skin necrosis(J). Postgraduate Medical Journal, 2021:postgradmedj-2021-139988]. Direct thrombin inhibitors (DTIs), as well as the anti-Factor Xa inhibitor, have been widely used in the clinical setting in recent years. Rivaroxaban is used to treat pulmonary embolism and deep vein thrombosis, and the drug is also used for secondary prevention of recurrent VTE in adults (5). This article evaluates the security and effectiveness of rivaroxaban vs. warfarin for the prevention of VTE.

## 2. Materials and methods

### 2.1. Study selection

We searched for studies published in EMBASE, Medline, PubMed, Web of Science, and the Cochrane Clinical Trials Database between January 2000 and October 2021, using the keywords: (1) rivaroxaban; (2) warfarin; (3) venous thromboembolism; and (4) bleeding. Combinations of keywords using the Boolean operators “and/or” were adopted for the search strategy. Two reviewers independently analyzed the literature, extracted and analyzed data.

The inclusion criteria were: (1) patients receiving rivaroxaban and warfarin; (2) primary endpoint outcome was VTE, (3) the full text of the article is written in English. The criteria for exclusion from the article were as follows: (1) Formatted as protocols, reviews, or letters; (2) articles found by duplicate search; (3) articles with unclear results.

### 2.2. Data extraction and synthesis

Two reviewers independently collected articles according to predetermined standards. In case of disagreement, it was resolved by negotiate with the third reviewer. The reviewers collected the following information from articles that met the criteria: Author name, country, year of publication, type of study, patient information (number, gender, age, treatment received), follow-up, and primary outcomes.

### 2.3. Quality assessment

The quality of the article literature was evaluated by the Cochrane risk of bias tool. Risk bias of non-randomized studies was evaluated using the Newcastle–Ottawa scale, which includes adequacy of comparability of studies, cohort selection, and assessment of outcomes.

### 2.4. Statistical analyses

The study was performed by STATA 13.0 and Revman 5.4. Risk ratios (RRs) were used for outcomes. Data heterogeneity was evaluated using *I*^2^ values: a fixed-effect model was used if *I*^2^ was <50%;and a random-effect model was used if *I*^2^ was ≥50%,; if *P* < 0.05 or *I*^2^ > 50%, the random effect model was used; if *P* ≥ 0.05 and *I*^2^ ≤ 50%, the fixed effect model was used for analysis. Sensitivity analysis was conducted by excluding merged studies individually and observing whether the result significantly changed. Publication bias was evaluated using funnel plots and Egger’s regression.

## 3. Results

### 3.1. Search process

**Figure 1:**
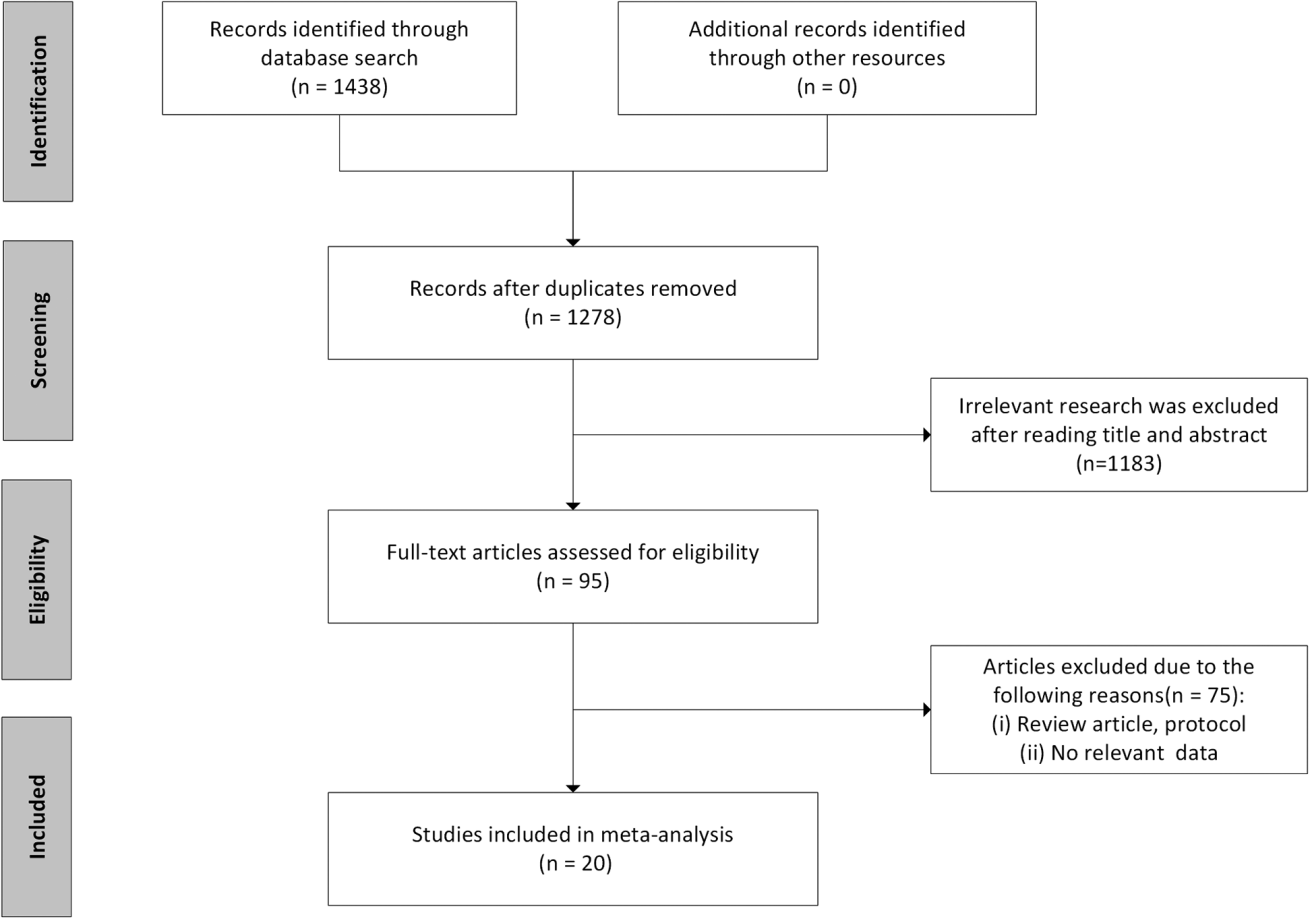
Flowchart of literature search and study selection.

According to PRISMA guidelines, 1,438 articles were collected by screening search strategies. After the removal of duplicates, 1,278 studys were retained, and 1,183 articles were excluded after screening the abstracts and titles. After reading the study content, 75 articles were further excluded. Ultimately, 20 eligible studies were included (Figure 1).

### 3.2. Characteristics of the included studies

The studies were published in the last 6 years (2017–2021) and included 1 RCT study, 1 prospective comparative study, and 18 retrospective comparative studies (Table 1). These 20 studies included a total of 230,320 patients, of which 74,018 received rivaroxaban and 156,302 received warfarin.

**Table 1:** Baseline characteristics of studies included in the meta-analysis.

Study	Country	Study design	Disease	Group	No. of patients	Gender (M/F)	Age*	Follow-up	Outcomes
Pengo 2018	Italy	RCT	Antiphospholipid syndrome	Rivaroxaban	59	20/39	46.5 ± 10.2	569 days	VTE, major bleeding, all-cause mortality
				Warfarin	61	23/38	46.1 ± 13.2		
Costa 2020a	United States	RCS	VTE	Rivaroxaban	2,097	914/1,183	50 (39, 62)	6-months	VTE, major bleeding
				Warfarin	2,842	1,239/1,603	51 (40, 64)		
Perales 2019	United States	RCS	Extreme obesity and high body weight	Rivaroxaban	84	44/40	56 ± 14	12-months	VTE, major bleeding, nonmajor bleeding, all-cause mortality
				Warfarin	92	51/41	55 ± 15		
Lullo 2018	Italy	RCS	Chronic kidney disease	Rivaroxaban	247	134/113	66.0 ± 4.4	16-months	VTE, major bleeding, nonmajor bleeding
				Warfarin	100	58/42	66.5 ± 4.6		
Laliberté 2014	Canada	RCS	Nonvalvular atrial fibrillation	Rivaroxaban	3,654	1,789/1,865	73.3 ± 8.4	6-months	VTE, major bleeding
				Warfarin	14,616	7,086/7,530	73.7 ± 8.3		
Russo-Alvarez 2018	United States	RCS	Nonvalvular atrial fibrillation	Rivaroxaban	472	289/183	73.6 ± 11.5	12-months	Major bleeding
				Warfarin	472	300/172	73.6 ± 11.9		
Coleman 2018a	United States	RCS	Unprovoked VTE	Rivaroxaban	10,489	5,653/4,836	56 (45, 64)	6-months	VTE, major bleeding
				Warfarin	26,364		56 (46, 65)		
Kushnir 2019	United States	RCS	Morbid obesity	Rivaroxaban	152	52/100	52.4 ± 14.7	>90 days	VTE, major bleeding
				Warfarin	167	49/118	58.1 ± 15.1		
Fung 2019	China	RCS	VTE	Rivaroxaban	90	37/53	63.3 ± 18.2	12-months	VTE, major bleeding, nonmajor bleeding
				Warfarin	91	43/47	61.8 ± 17.9		
Coleman 2018b	United States	RCS	Provoked VTE	Rivaroxaban	4,454	2,165/2,289	57 (47, 65)	6-months	VTE, major bleeding
				Warfarin	13,164	6,437/6,727	59 (48, 69)		
Coleman 2018c	United States	RCS	Frail patients	Rivaroxaban	1,365	470/895	81.8 ± 6.4	12-months	VTE, major bleeding
				Warfarin	5,504	1,959/3545	82.4 ± 6.3		
Shah 2018	United States	RCS	Cancer and atrial fibrillation	Rivaroxaban	2,808	1,665/1,143	73.8 ± 10.2	11-months	VTE, major bleeding
				Warfarin	10,021	6,073/3948	75.4 ± 10.1		
Coleman 2018d	United States	RCS	VTE with a hypercoagulable state	Rivaroxaban	403	201/202	50.3 ± 14.5	6-months	VTE, major bleeding
				Warfarin	403	204/199	50.3 ± 14.5		
Larsen 2017	Denmark	PCS	Unprovoked VTE	Rivaroxaban	1,751	958/753	62.6 ± 17.4	6-months	VTE, major bleeding, all-cause mortality
				Warfarin	3,253	1,770/1,473	62.6 ± 17.0		
Coleman 2017	United States	RCS	VTE	Rivaroxaban	13,609	7,081/6,258	NR	12-months	VTE, major bleeding
				Warfarin	32,244	16,220/16,024	NR		
Costa 2020b	United States	RCS	Obese patients with acute VTE	Rivaroxaban	6,755	3,131/3,624	NR	12-months	VTE, major bleeding
				Warfarin	6,755	3,060/3,695	NR		
Streiff 2018	United States	RCS	Cancer with VTE	Rivaroxaban	892	458/434	73.4 ± 10.2	12-months	VTE, major bleeding
				Warfarin	876	452/424	73.3 ± 9.3		
Costa 2020c	United States	RCS	Nonvalvular atrial fibrillation or VTE	Rivaroxaban	683	253/430	NR	12-months	VTE, major bleeding
				Warfarin	683	245/438	NR		
Roetker 2018	United States	RCS	VTE	Rivaroxaban	21,064	10,679/10,385	59 ± 16	12-months	All-cause mortality
				Warfarin	35,704	17,245/18,459	64 ± 16		
Spyropoulos 2019	United States	RCS	Morbidly obese patients with VTE	Rivaroxaban	2,890	1,141/1,749	53.3 ± 12.9	12-months	VTE, major bleeding
				Warfarin	2,890	1,150/1,740	53.1 ± 13.1		

BMI, body mass index; RCT, randomized controlled trial; RCS, retrospective cohort study; PCS, prospective cohort study; VTE, venous thromboembolism; NR, not reported.

*Data are presented as mean ± SD values, median (interquartile range) values.

### 3.3. Quality assessment results

The Cochrane bias risk assessment or Newcastle–Ottawa Scale were used to evaluate the quality of the studies (Table 2).

**Table 2:** Assessment of methodological quality of included studies.

Randomized controlled trial									
Study	Random allocation	Hidden distribution	Blind method	Incomplete outcome data	Selective reporting of results	Other bias	Quality level		
Pengo 2018	Low risk	Low risk	Low risk	Low risk	Low risk	Low risk	High		
Cohort study									
Study	Selection				Comparability of cohorts	Outcomes			Score
	Representativeness of cohort	Selection of nonexposed cohort	Ascertainment of exposure	Outcome lacking at tde beginning		Outcome assessment	Sufficient follow-up time	Follow up adequacy	
Costa 2020a	★	★	★	★	★⋆	★	★	★	8
Perales 2019	★	★	★	★	★★	★	★	★	9
Lullo 2018	★	★	★	★	★⋆	⋆	★	⋆	6
Laliberté 2014	★	⋆	★	⋆	★★	★	★	★	7
Russo-Alvarez 2018	★	⋆	★	★	★⋆	★	⋆	★	6
Coleman 2018a	★	★	★	⋆	★★	★	★	★	8
Kushnir 2019	★	★	★	★	★★	★	⋆	★	8
Fung 2019	★	★	★	★	★★	★	★	★	9
Coleman 2018b	★	★	★	★	★★	⋆	★	★	8
Coleman 2018c	★	★	★	★	★★	⋆	★	★	8
Shah 2018	★	⋆	★	★	★⋆	★	★	★	7
Coleman 2018d	★	★	★	★	★★	⋆	★	★	8
Larsen 2017	★	★	★	★	★★	★	★	★	9
Coleman 2017	★	★	★	⋆	★⋆	★	★	★	7
Costa 2020b	★	★	★	★	★★	⋆	★	★	8
Streiff 2018	★	★	★	★	★⋆	★	★	⋆	7
Costa 2020c	★	★	★	★	★⋆	★	★	★	8
Roetker 2018	★	★	★	⋆	★⋆	★	★	⋆	6
Spyropoulos 2019	★	★	★	★	★⋆	★	★	★	8

### 3.4. Results of the meta-analysis for outcomes

#### 3.4.1. Incidence of VTE

**Figure 2:**
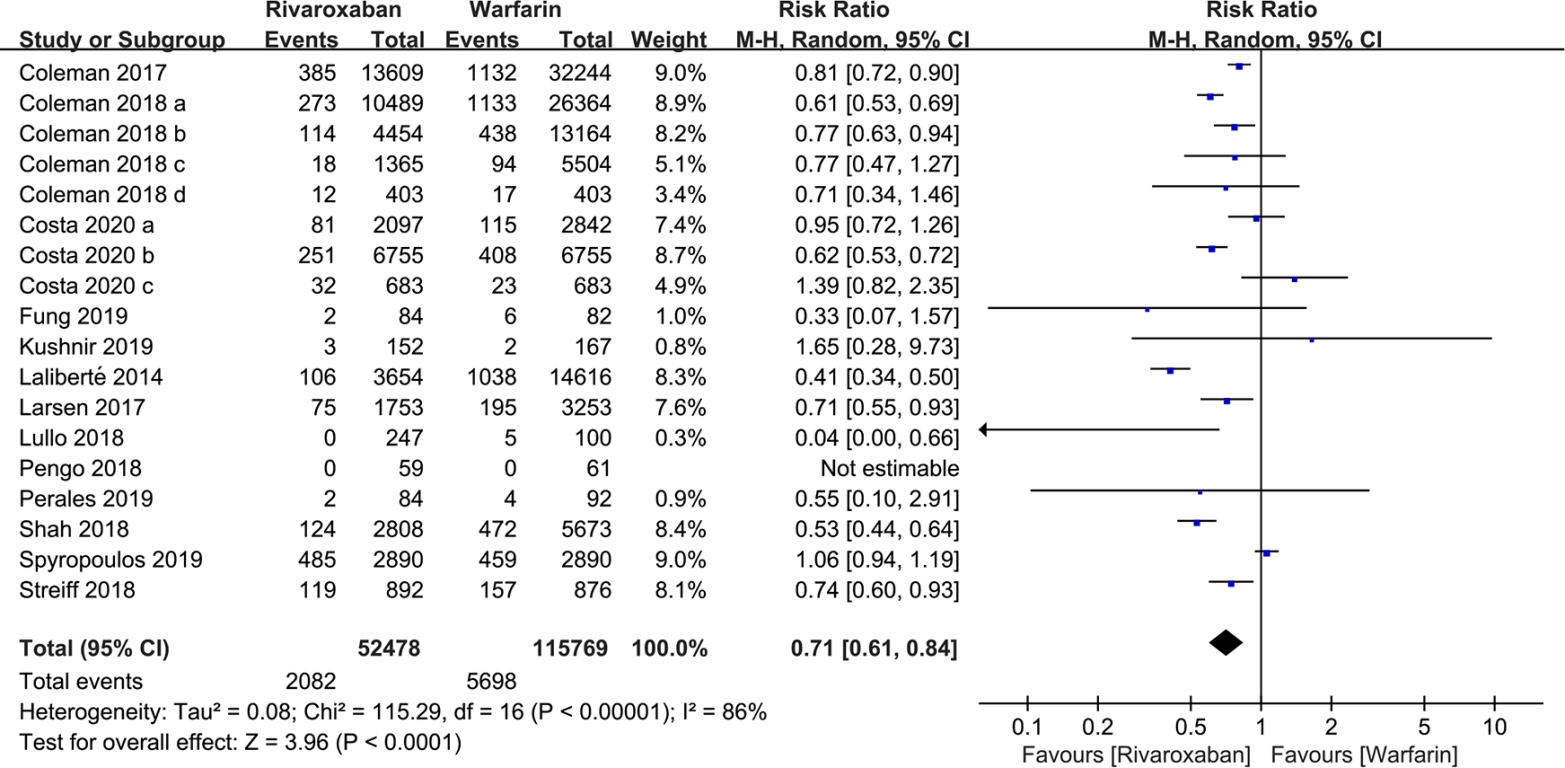
Forest plot for venous thromboembolism (VTE) between rivaroxaban group and warfarin group.

**Figure 3:**
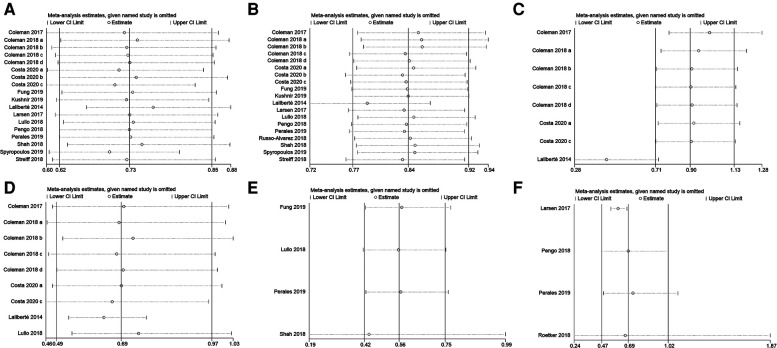
Sensitivity analysis of (**A**) VTE, (**B**) major bleeding, (**C**) intracranial hemorrhage, (**D**) gastrointestinal bleeding, (**E**) nonmajor bleeding, and (**F**) all-cause mortality.

A total of 18 studies with 168,247 patients reported the incidence of VTE. According to the pooled estimate, compared to the warfarin, the rivaroxaban group had a significantly lower incidence of VTE (RR: 0.71, 95% confidence interval (CI) [0.61, 0.84]; *P* < 0.0001, random effect model), with significant heterogeneity among the included studies (*I*^2^ = 86%, *P* < 0.0001) (Figure 2). Based on the results of the sensitivity analysis, ignoring any single study would not affect the final results (Figure 3).

#### 3.4.2. Major bleeding

**Figure 4:**
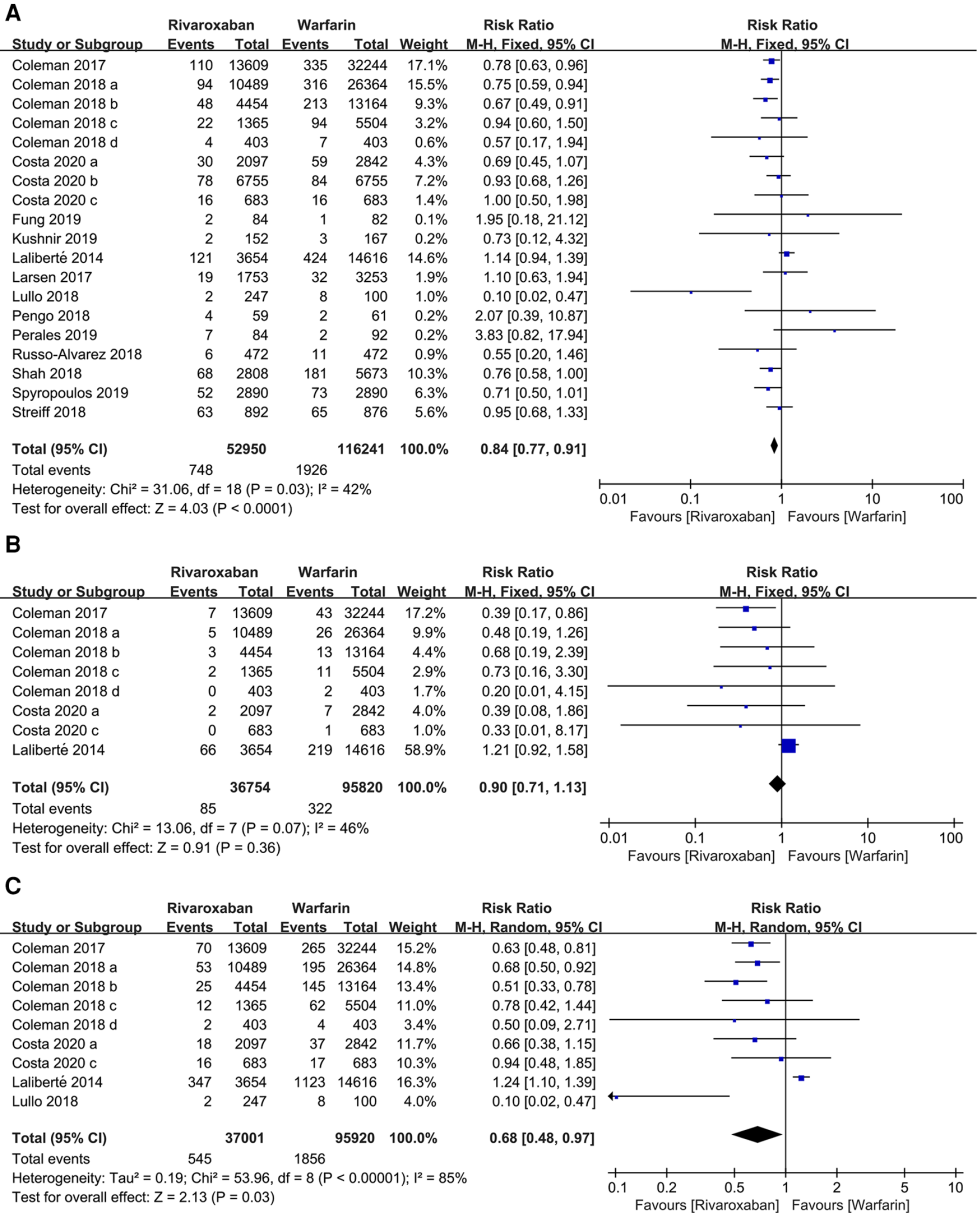
Forest plot for major bleeding between rivaroxaban group and warfarin group. (**A**) major bleeding, (**B**) intracranial hemorrhage, (**C**) gastrointestinal bleeding.

169, 191 patients in 19 studies were reported major bleeding. According to the forest plot, the rivaroxaban group was lower than the warfarin group in the incidence of major bleeding [RR: 0.84, 95% CI (0.77, 0.91); *P* < 0.0001, fixed effect model], without significant heterogeneity among the included studies (*I*^2^ = 42%, *P* = 0.03) (Figure 4). The result was not changed after the sensitivity analysis (Figure 3).

Subgroup analyses were performed according to the site of major bleeding. The pooled results of the incidence of intracranial hemorrhage and gastrointestinal bleeding are presented in Figure 4. There was no significant difference in the incidence of intracranial hemorrhage between the two groups [RR: 0.90, 95% CI (0.71, 1.13); *P* = 0.36, fixed effect model], while the incidence of gastrointestinal bleeding in the rivaroxaban group was significantly lower than the warfarin group [RR: 0.68, 95% CI (0.48, 0.97); *P* = 0.03, random effect model] (Figure 4). The sensitivity analysis showed that the study by Laliberté et al. had a significant impact on the pooled results of the incidence of intracranial hemorrhage when it was removed (6) (Figure 3).

#### 3.4.3. Nonmajor bleeding

**Figure 5:**
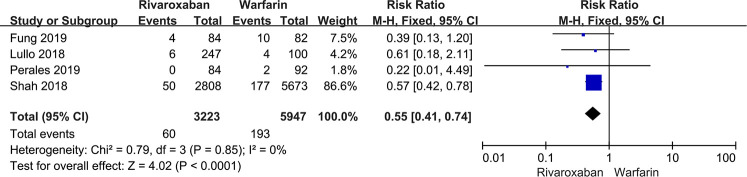
Forest plot for nonmajor bleeding between rivaroxaban group and warfarin group.

4 studies contained information regarding nonmajor bleeding events. The results showed that rivaroxaban decreased the incidence of nonmajor bleeding events compared with warfarin [RR: 0.55, 95% CI (0.41, 0.74); *P* < 0.0001, fixed effect model], without significant heterogeneity (*I*^2^ = 0%, *P* < 0.0001) (Figure 5). The result was not changed after the sensitivity analysis (Figure 3).

#### 3.4.4. All-cause mortality

**Figure 6:**
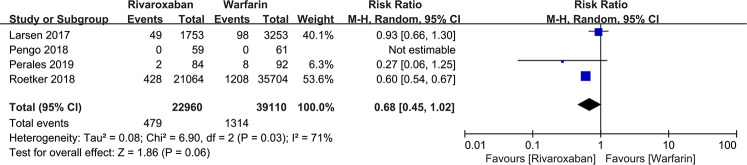
Forest plot for all-cause mortality between rivaroxaban group and warfarin group.

Four studies mentioned all-cause mortality. A total of 1,793 out of 62,070 patients in the two groups died from various causes. These results showed no significant difference in all-cause mortality between the rivaroxaban and warfarin groups [RR: 0.68, 95% CI (0.45, 1.02); *P* = 0.06, fixed effect model], and no significant heterogeneity among the studies (*I*2 = 0%, *P* = 0.85) (Figure 6). The sensitivity analysis showed that the result was relatively stable (Figure 3).

#### 3.4.5. Publication bias

**Figure 7:**
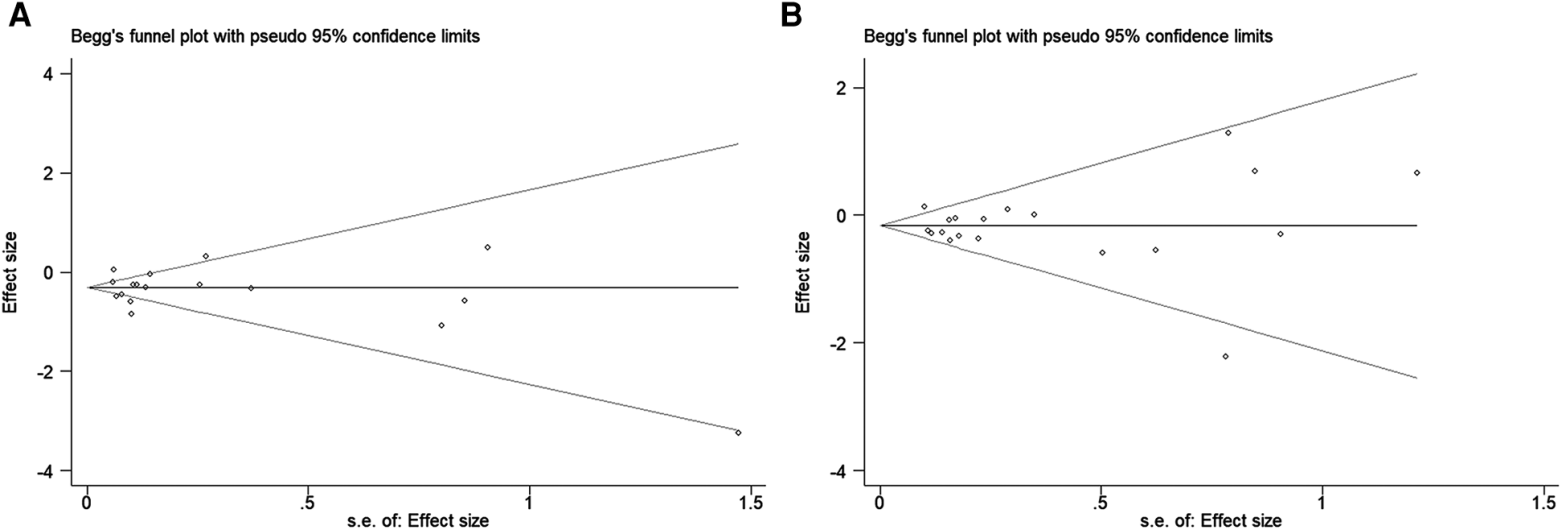
Funnel plot of (**A**) VTE and (**B**) major bleeding.

There were two funnels, which are basically symmetrical (Figure 7). According to Egger's linear regression quantitative assessment, no significant publication bias in this study (VTE, *P* = 0.617; major bleeding, *P* = 0.821).

## 4. Discussion

VTE is a serious complication that has a major impact on the normal of life of patients (7). Since 1941, warfarin has been a commonly used anticoagulant in the clinic (8). Warfarin decreases INR levels and increases the risk of bleeding, and frequent testing of coagulation is required while taking warfarin (9). Rivaroxaban overcomes the limitations of traditional anticoagulants in the prevention or treatment of arteriovenous thromboembolism. As a factor of Xa inhibitor, rivaroxaban can be taken orally, with a benifit of rapid onset of action and low adverse effects (10–13). This study investigates the safety and efficacy of rivaroxaban vs. warfarin in VTE prevention for the first time. According to the present study, rivaroxaban was significantly superior to warfarin in VTE prevention [RR: 0.71, 95% CI (0.61, 0.84); *P* < 0.0001]. Warfarin, a commonly used oral anticoagulant, is principally metabolized by CYP2C9 to form 7-hydroxywarfarin. New oral anticoagulants targeting Factor Xa, such as apixaban, rivaroxaban, and edoxaban, have been approved and will become preferred treatments for VTE. In this meta-analysis, the use of rivaroxaban resulted in a lower incidence of VTE than warfarin.

This study has implications for VTE prevention: (1) 20 studies (1 RCT, 1 prospective comparative, and 18 retrospective comparative studies) were retrieved. A large number of patients were included in this study (230,320 participants); (2) there is no previous meta-analyses of the effects of rivaroxaban vs. warfarin. Moreover, as a novel anticoagulant drug, rivaroxaban has no antagonist, so its safety comparison with traditional anticoagulant drugs is of more concern to clinicians, the ability of major or non-major bleeding to cause mortality was included in this study to evaluate the safety; (3) VTE was used to evaluate the drug efficacy, so this study has a high precision.

The limitations of this study are: (1) Only English language literatures were included in this study, so there may be a selection bias; (2) the baseline characteristics of the participants, such as chronic diseases, tumors, trauma, etc., were not considered, which might potentially affect the outcomes of the study; (3) The use of a non-major bleeding definition to count bleeding complications other than major bleeding was not specific enough, which may potentially affect the results of the study; (4) Due to the limitation in the descriptions of the cases treated in the major trials we have focussed on, no subgroup analysis of the incidence of VTE was performed in this study.

To summarize, this meta-analysis revealed that rivaroxaban is superior to warfarin in preventing VTE. Encouraging as our data analysis is there remains a need for further, more optimally designed trials to confirm the trend that is evident in the studies published todate.

## Data Availability

The original contributions presented in the study are included in the article/Supplementary Material, further inquiries can be directed to the corresponding author/s.
